# The heterogeneous nature of atrioventricular conduction tissues in tetralogy of Fallot demonstrated by hierarchical phase-contrast tomography

**DOI:** 10.1016/j.xjse.2026.100111

**Published:** 2026-03-20

**Authors:** Vaishnavi Sabarigirivasan, Joseph Brunet, Hector Dejea, Adrian Crucean, Anusha Jegatheeswaran, Claudio Capelli, Endrit Pajaziti, Theresa Urban, Joanna Purzycka, Paul Tafforeau, Claire L. Walsh, Peter D. Lee, Andrew C. Cook

**Affiliations:** a Institute of Cardiovascular Sciences, University College London, London, United Kingdom; b Department of Mechanical Engineering, University College London, London, United Kingdom; c European Synchrotron Radiation Facility, Grenoble, France; d Birmingham Children's Hospital, Birmingham, United Kingdom; e Great Ormond Street Hospital for Children NHS Trust, London, United Kingdom; f Research Complex at Harwell, Rutherford Appleton Laboratory, Didcot, United Kingdom

**Keywords:** tetralogy of Fallot, HiP-CT, right bundle branch, arrhythmia

## Abstract

**Objectives:**

Postoperative arrhythmias are frequent after tetralogy of Fallot (ToF) repair, yet anatomic substrate and preventive strategies remain poorly defined. Using hierarchical phase-contrast tomography (HiP-CT), the atrioventricular conduction system in pediatric ToF specimens was investigated nondestructively and in 3-dimensions (3D).

**Methods:**

Eighteen whole-heart specimens (11 ToF, 7 controls) were imaged at the European Synchrotron Radiation Facility. Segmentation and 3D renderings demonstrated gross morphology. Morphology, size, depth, and course of the nonbranching bundle and right bundle branch (RBB) were quantified using custom computational pipelines. Segmentations were visualized in VheaRts, a Unity3D-based XR platform.

**Results:**

The ToF conduction system was more draped than in controls, bilaterally spanning the septum, resembling early embryonic architecture. The RBB was variable in origin, course, and morphologic structure with significantly smaller indexed cross-sectional area in native ToF versus controls (0.05 ± 0.20 vs 0.50 ± 0.20 mm^2^, *P* = .005). One anomalous fasciculo-ventricular connection and 6 dead-end tracts were found. Regions at surgical risk included the posterior-inferior margin of the ventricular septal defect and the septal crest along its nadir. The superior margin of the ventricular septal defect at its intersection with the aortic root was free of conduction tissue. The HiP-CT to virtual reality pipeline enabled interactive 3D visualization of conduction pathways relative to key structures.

**Conclusions:**

This first pediatric cardiac HiP-CT series reveals a broader anomalous conduction complex in ToF, including variable RBB origin and hypoplasia, providing insight into preoperative vulnerability, arrhythmia, and surgical risk.

## Graphical Abstract

**Figure:**
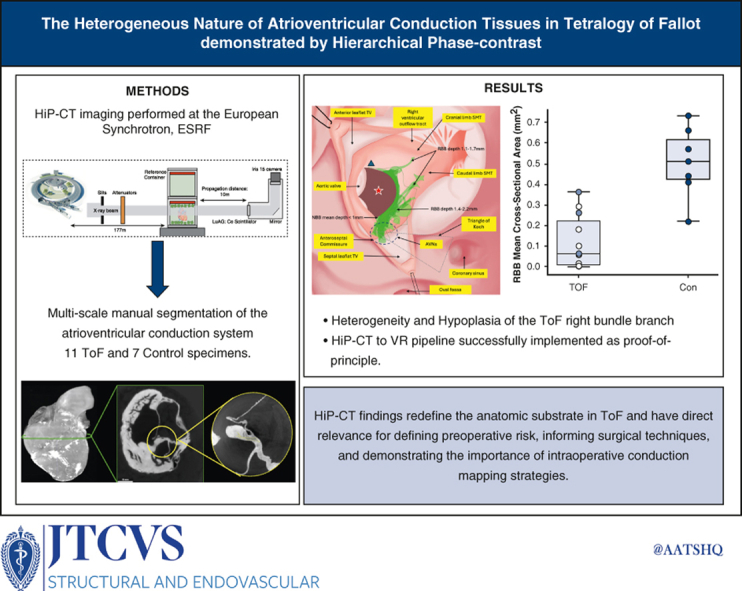

**Figure:**
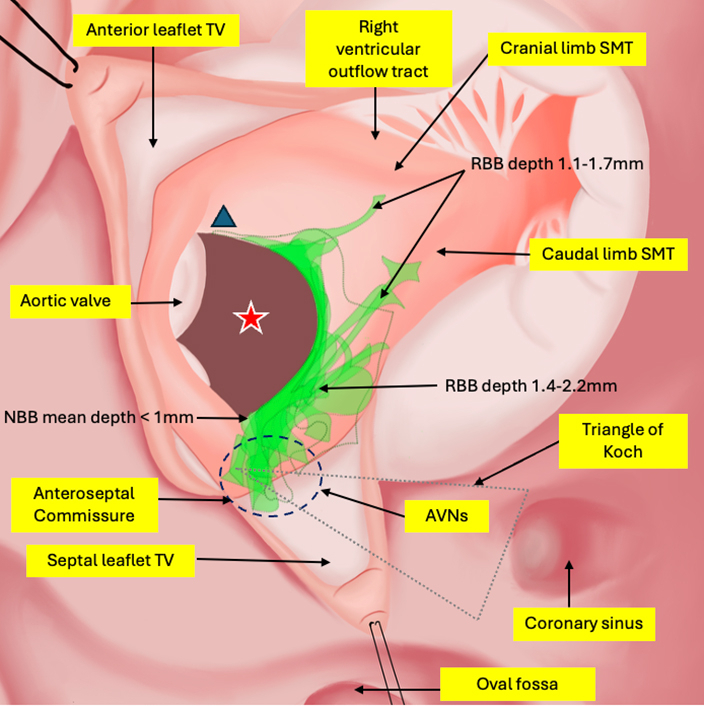
Surgical view of TOF right bundle branches with depth and key landmarks marked.

**Central Message**

Hierarchical phase-contrast tomography reveals atrioventricular conduction system heterogeneity in Tetralogy of Fallot, providing insights into arrhythmia risk and guiding safe suture placement.

**Perspective**

Postoperative arrhythmias are frequent in Tetralogy of Fallot. This study is the first to explore pediatric anatomical specimens using Hierarchical phase-contrast tomography. This non-destructive technique can reveal the 3D nature of the conduction system in whole-heart specimens, providing novel anatomic insights and surgical guidance.

Postoperative arrhythmias represent a significant complication in tetralogy of Fallot (ToF), with reported incidences reaching 43% in some multicenter studies.^1^^,^^2^ Ventricular arrhythmias and conduction delays influence transient and long-term morbidity and mortality, but their etiology remains incompletely understood. Postoperative arrhythmias may result from arrhythmogenic changes in the atrioventricular conduction system (AVCS) or its surrounding myocardium, causing junctional ectopic tachycardia, or from direct injury during ventricular septal defect (VSD) closure or outflow tract resection.^1^^,^^2^ Preoperative right bundle branch (RBB) block is rare (∼4%) but occurs in up to 60% of patients at discharge postoperatively. Its prevalence increases to greater than 80% in childhood and adolesence.^3^

Current knowledge of the ToF AVCS system is based on seminal work in the 1950s to 1980s, which used destructive dissection and histologic serial sectioning of the “conduction block” in small numbers of pediatric heart specimens.^4^, ^5^, ^6^, ^7^, ^8^, ^9^, ^10^, ^11^, ^12^ Although this laid the basis for surgical intervention, no additional data have been generated because of the lack of novel, nondestructive techniques. Hierarchical phase-contrast tomography (HiP-CT) can be used to image intact human organs with exceptional resolution using the phase-contrast capabilities of the BM18 beamline at the European Synchrotron Radiation Facility (ESRF) Extremely Brilliant Source.^13^ It allows the nondestructive examination of whole organs, with a local zoom down to histologic levels (2 μm voxel size), eliminating the need for physical subsampling.^13^ Studies using other synchrotron imaging techniques or protocols have visualized the AVCS in whole fetal and pediatric specimens,^14^, ^15^, ^16^ but only HiP-CT has been able to achieve this in intact adult hearts.^17^ These investigations have been restricted to at most 2 specimens per study. We present the first whole-organ pediatric cardiac disease case series using HiP-CT. An overview of the study design is shown in Figure 1.

**Figure 1:**
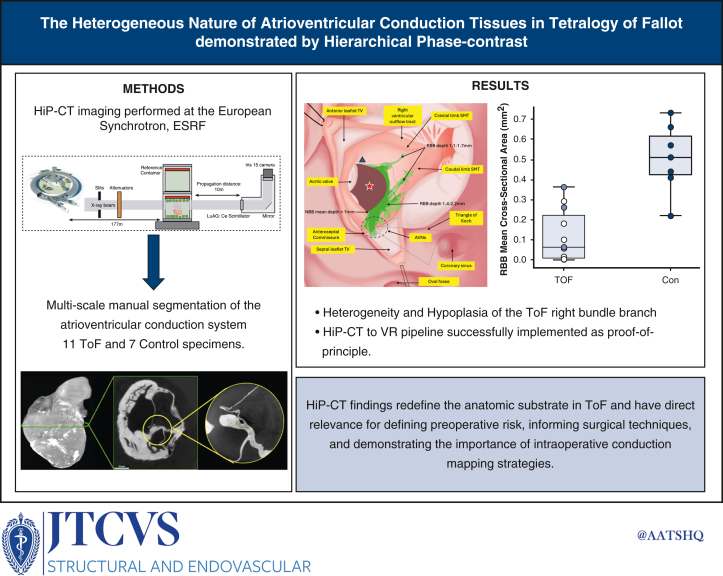
Hierarchical phase-contrast tomography demonstrates the heterogenous nature of atrioventricular conduction tissues in tetralogy of Fallot.

## Methods

### Specimen Description

Five hundred and fifty ToF hearts from the University College London (UCL) Cardiac Archive Biobank (UK Human Tissue Authority Research License 12220) were reviewed and 11 were selected for imaging (Table 1). Hearts were chosen if they were within specific size constraints (105 mm × 105 mm × 167 mm), were in good condition on visual inspection, and possessed intact right ventricular outflow tracts and VSDs. Seven structurally normal control specimens from Birmingham Children's Hospital (BCH) (ethical approval: REC 22/PR/0906) and UCL, matched to ventricular size, were selected using the aforementioned criteria. All specimens were assigned a unique ID number under UCL CAB and transported to ESRF under transport authorization no. IE-2023-2966, issued by the French Ministry of Higher Education and Research. This study is of archival postmortem material. In the United Kingdom, there has always been a requirement for consent for postmortem examination including use for “research and education.” Many of these samples are now older than 60 to 80 years, and it is not considered appropriate for us to ask families for consent for publication in retrospect. Instead, families are given the opportunity to withdraw consent for use of material at any time.

**Table 1:** Morphologic characteristics of specimens included in this study

ID	Short-form link	Specimen morphology
UCL-ZCR-125	T1	PM outlet VSD, pulmonary stenosis
UCL-ZCR-173	T2	PM outlet VSD, absent pulmonary valve
UCL-ZCR-185	T3	PM outlet VSD, pulmonary atresia
UCL-ZCR-450	T4	PM and juxta-arterial VSD, pulmonary stenosis
UCL-ZCR-727	T5	PM outlet VSD, pulmonary stenosis, repair - VSD closure + pericardial transannular patch
UCL-ZCR-769	T6	PM outlet VSD, absent pulmonary valve
UCL-ZCR-1770	T7	PM outlet VSD, pulmonary stenosis
UCL-ZCR-1771	T8	PM outlet VSD, pulmonary stenosis
UCL-ZCR-1840	T9	PM outlet VSD, pulmonary stenosis, repair - VSD closure + Dacron transannular patch
UCL-ZCR-2010	T10	PM outlet VSD, pulmonary stenosis, repair - VSD closure+ Dacron transannular patch
UCL-ZCR-2577	T11	Muscular outlet VSD, pulmonary atresia
UCL-ZCR-2359	C1	Control - normally connected heart
UCL-ZCR-2337	C2	Control - normall connected heart
UCL-ZCR-2625	C3	Control - normally connected heart
UCL-ZCR-2627	C4	Control - normall connected heart
UCL-ZCR-2628	C5	Control - normally connected heart
UCL-ZCR-2628	C6	Control - normally connected heart
UCL-ZCR-2630	C7	Control - normally connected heart

*UCL*, University College London; *ZCR*, Zayed Centre for Research; *PM*, perimembranous; *VSD*, ventricular septal defect.

The use of such material in the United Kingdom is regulated under the UK Human Tissue Act 2006 as “existing material” (research license no: 12220 [UCL]; postmortem license: 12,132 [BCH]). We have confirmed that publication, including images if anonymized, is allowed under UK Human Tissue Authority legislation.

The scanning of archival material, as in the current study, has been reviewed by Professor Sir Ian Kennedy QC, a leading British academic, lawyer, and expert in health law, ethics, and policy. His report recommended that such nondestructive investigations, with an aim to publish, should continue on such material under local governance. Research at each institution is governed by (1) local archive committees, (2) local Human Tissue Authority committees and (3) institutional Human Tissue Authority governance, and (4) through monitoring of institutional compliance by the UK Human Tissue Authority at regular inspections. Our last inspections were in 2024 (UCL) and 2025 (BCH), respectively. Institutional review board approval was not required.

### Sample Preparation and Data Acquisition

HiP-CT imaging was performed at beamline BM18 (ESRF)^18^ using a protocol modified from Walsh and colleagues^13^ and Brunet and colleagues.^19^ Details can be found in the Appendix E1.

### Data Processing and Segmentation

**Figure 2:**
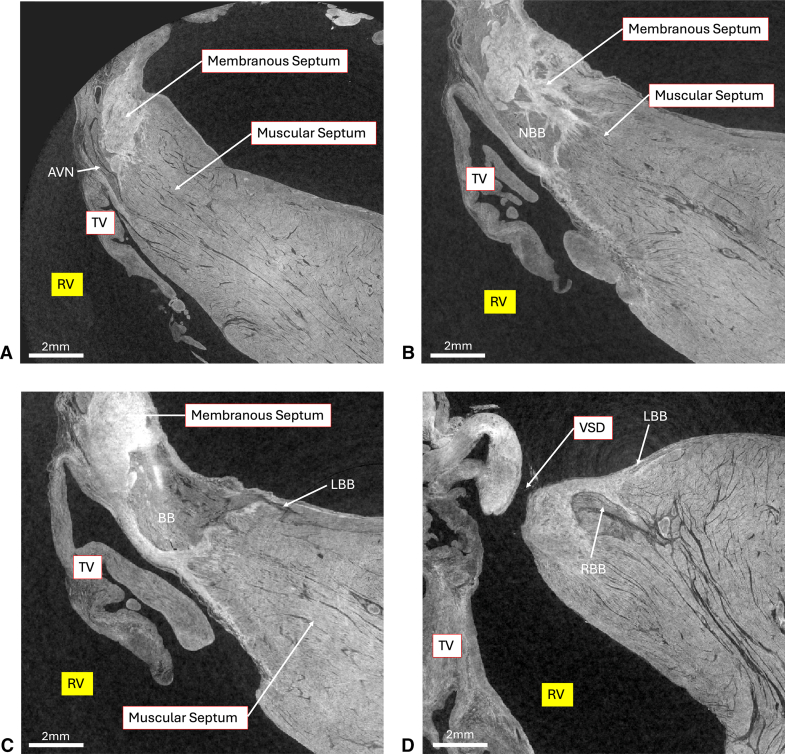
HiP-CT images of a ToF specimen with PM VSD and pulmonary stenosis in long-axis view. A, Atrioventricular node is visualized. B, First point where the nonbranching bundle (NBB) is completely ensheathed in fibrous tissue. C, Left bundle branch (LBB) appears from branching bundle (BB). D, Bifurcation of the right bundle branch (RBB) and LBB. *ToF*, Tetralogy of Fallot; *PM*, perimembranous; *VSD*, ventricular septal defect; *HiP-CT*, Hierarchical phase-contrast tomography; *TV*, tricuspid valve; *RV*, right ventricle.

Data reconstruction followed HiP-CT processing protocol as described by Brunet and colleagues.^19^ Manual segmentation was performed using Amira 2023.2 software. Figure 2 demonstrates the features of the AVCS as shown by HiP-CT. Details can be found in the Appendix E1.

### Virtual Reality (VR)

Whole-heart and conduction segmentations were exported separately from Amira (version 2023.2) in.obj (wavefront) format and downsampled using a custom Python script to minimize mesh complexity and reach a size below 200 GB (Code Availability). Exploration, visualization, and manipulation of 3-dimensional (3D) models was performed in VheaRts, an extended reality (XR) application developed in Unity3D using OpenXR framework, currently used with Meta Quest devices.^20^^,^^21^

### Morphometric Characteristics of the Nonbranching Bundle (NBB) and Right Bundle Branch (RBB)

NBB depth and relationship to the membranous septum remnant were quantified using Neuroglancer.^22^ RBB depth and cross-sectional area through its course were quantified using an in-house Python pipeline (see “Code Availability”). Control and ToF samples were compared using independent *t* tests.

### Code Availability

All analysis and utility scripts used in this study are openly available at the public repository: https://github.com/VaishnaviSabari/conduction-tools.git.

#### Data Availability

All reconstructed volumes are openly accessible through the Human Organ Atlas repository (https://human-organ-atlas.fr). Dataset DOIs corresponding to each donor are detailed in Table E1.

## Results

### Hierarchical Imaging

In whole-organ scans, full visualization of the AVCS was achieved in 4 of 7 control and 2 of 11 ToF specimens. The RBB was not distinguishable in the remaining datasets. In zoom scans, the atrioventricular node (AVN), NBB, branching bundle (BB), left bundle branch (LBB), and RBB origin could be visualized in 9 ToF specimens (Table E2).

### The AVCS in the Normal Heart

**Figure 3:**
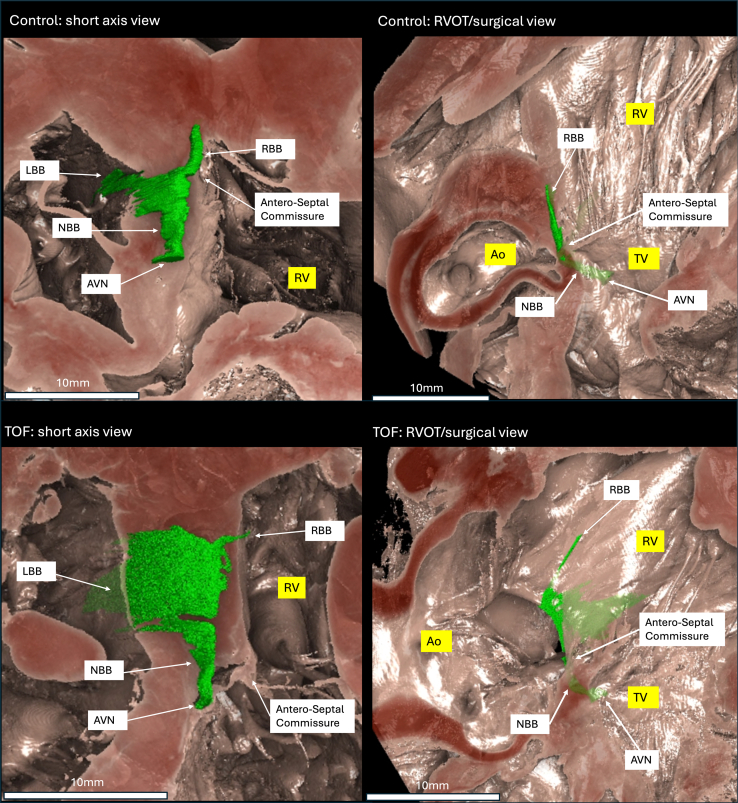
The conduction system in a control specimen in short axis and RVOT views and in a ToF specimen. *RVOT*, Right ventricular outflow tract; *ToF*, tetralogy of Fallot; *LBB*, left bundle branch; *RBB*, right bundle branch; *NBB*, nonbranching bundle; *AVN*, atrioventricular node; *RV*, right ventricle; *Ao*, aorta; *TV*, tricuspid valve.

The AVN was positioned at the apex of the triangle of Koch, at the anteroseptal commissure. The NBB coursed superiorly and centrally on the crest of the interventricular septum (IVS) and was aligned with the anteroseptal commissure (ASC). The LBB originated first, and the RBB arose distal to and within 2 mm of the tricuspid valve (TV) annulus. The RBB quickly penetrated the myocardium and remained closely associated with the inferior limb of the septomarginal trabeculation as it extended inferiorly toward the medial papillary muscle (MPM) or cords. The LBB exhibited variable morphology; sheet-like in some, and narrower in others. Overall, the LBB was relatively compact and positioned subendocardially (Figure 3).

### The Conduction System in ToF

The ToF AVCS exhibited significant heterogeneity. The AVN remained at the apex of the triangle of Koch, reflecting correct alignment of the atrial and ventricular septa. The NBB deviated to the left side of the IVS in 4 specimens (T7, T8, T10, and T11), to the right in 1 specimen (T1), and in 4 specimens it was central (T3-T6). In 2 specimens (T2 and T9), the NBB region was fragmented. In perimembranous VSD samples, the NBB was positioned between the membranous and muscular septa. The NBB originated on the atrial side of the TV annulus in 6 specimens (T2-4, T7, T9, and T11), aligned with the ASC in 2 specimens (T1 and T8), and was on the ventricular side in 3 specimens (T5, T6, and T10). In the inferior corner of the perimembranous (PM) VSD (the region of mitral-tricuspid continuity), the NBB was less than 1 mm below the endocardial surface (0.12 ± 0.09 mm [median, 0.09; range, 0.005-0.30]). At the TV hinge line, conduction tissue lay deeper: average depth of 0.49 ± 0.30 mm (median, 0.40; range, 0.20-1.27).

Dead-end tracts were found in 6 specimens (T1-3, T6, T9, and T10), resembling a “saddle-shaped” extension, following the VSD contour. One large fasciculoventricular tract was found (T7).

**Figure 4:**
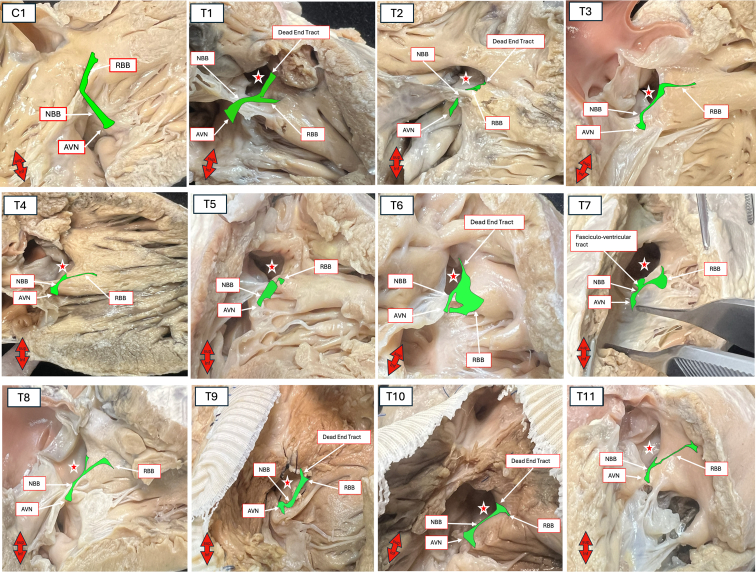
Atrioventricular conduction system represented on a control specimen and ToF specimens. *Red star* shows the ventricular septal defect. Compass demonstrates the image orientation: inferior points toward diaphragmatic surface of the heart. *ToF*, Tetralogy of Fallot; *RBB*, right bundle branch; *NBB*, nonbranching bundle; *AVN*, atrioventricular node.

**Figure 5:**
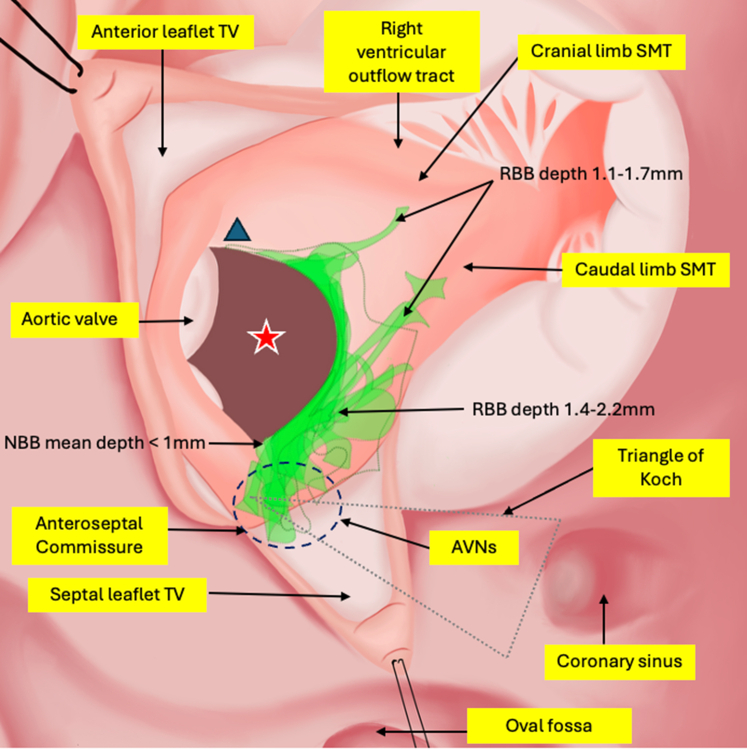
Representation of all ToF RBBs in this study from surgical view with the depth at relevant locations noted. *Red star* shows the ventricular septal defect, the *navy triangle* shows the conduction-free zone. *ToF*, Tetralogy of Fallot; *RBB*, right bundle branch; *TV*, tricuspid valve; *SMT*, septomarginal trabeculation; *NBB*, nonbranching bundle; *AVN*, atrioventricular node.

**Figure 6:**
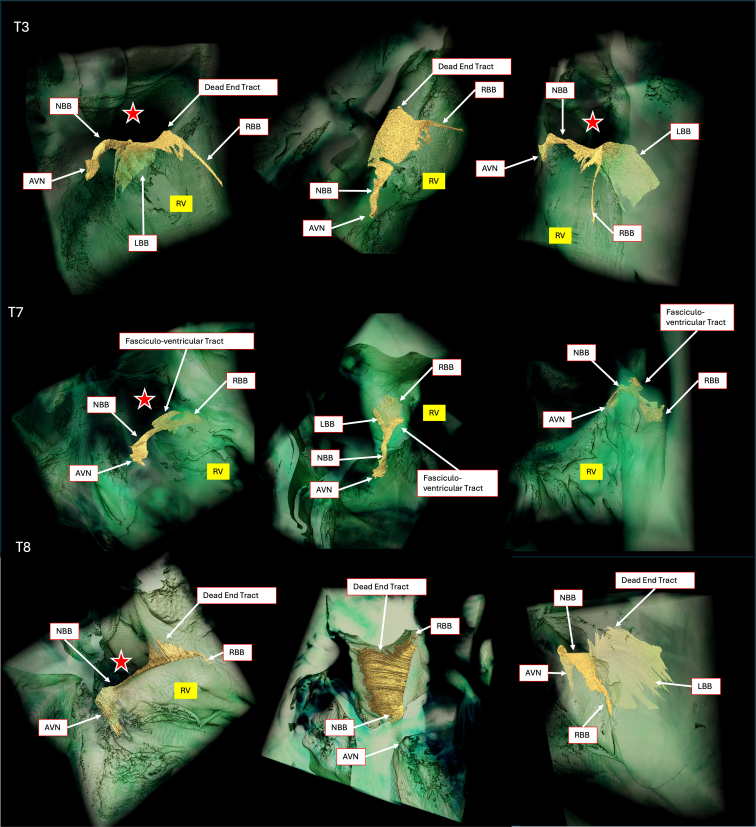
Three-dimensional representations of the atrioventricular conduction system in 3 ToF samples. *Red star* shows the ventricular septal defect. *ToF*, Tetralogy of Fallot; *NBB*, nonbranching bundle; *AVN*, atrioventricular node; *RBB*, right bundle branch; *LBB*, left bundle branch; *RV*, right ventricle.

**Figure E1:**
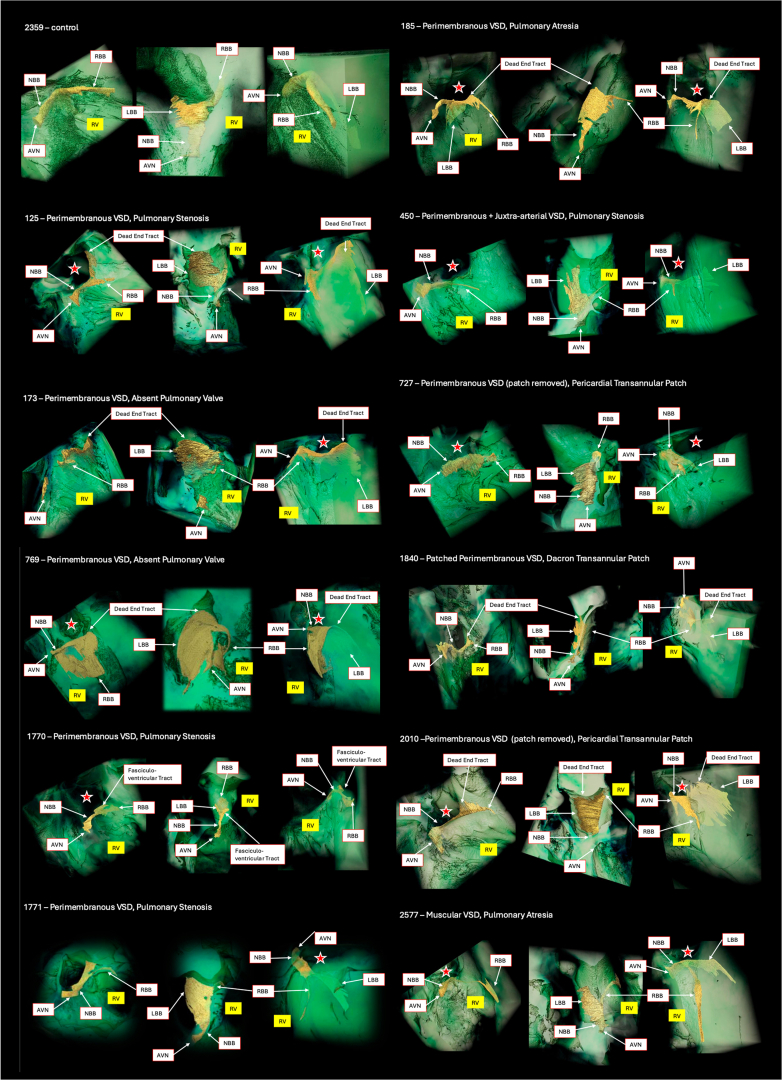
Three-dimensional renderings of conduction system segmentations in 1 control and all ToF specimens. *Red star* shows the VSD. *ToF*, Tetralogy of Fallot; *VSD*, ventricular septal defect; *AVN*, atrioventricular node; *NBB*, nonbranching bundle; *RBB*, right bundle branch; *LBB*, left bundle branch; *RV*, right ventricle.

In 4 specimens (T1, T2, T4, and T6), the RBB emerged closely associated with the ASC; in the rest, it branched later without a distinctive anatomical landmark. The RBB course was associated with the MPM in 6 of 11 specimens (T1-3, T7, T8, and T11) and the remainder coursed inferior to the caudal limb of septomarginal trabeculation, unrelated to the MPM. The RBB was broad in 2 samples (T6 and T7), draping extensively either over the right side of the septal crest or fanning bilaterally over the septal crest; whereas in another, it was crescent-shaped (T5). Representations of the ToF AVCS are found in Figures 4 and 5. 3D renderings are found in Figure 6, Video 1, and Figure E1.

### Association of Conduction Tissue With the Membranous Remnant of the IVS

In PM VSDs, the mean height of the membranous septum remnant was 0.9 ± 0.19 mm. In 3 specimens (T4, T5, and T7), conduction tissue penetrated the muscular septum below the level of the VSD, leaving the membranous septum and surrounding regions free of conduction tissue. In the remaining samples and in the muscular VSD case, conduction tissue ran between the membranous and muscular septa.

### Morphometric Characteristics of the RBB

In normal samples, the RBB average depth throughout its course ranged between 0.6 and 1.2 mm. The RBB became more superficial and then penetrated the myocardium approximately 1 mm from its origin. In ToF specimens, the RBB was deeper, ranging from 1 to 2.2 mm, but showed variability throughout its course (Figure 5).

**Figure 7:**
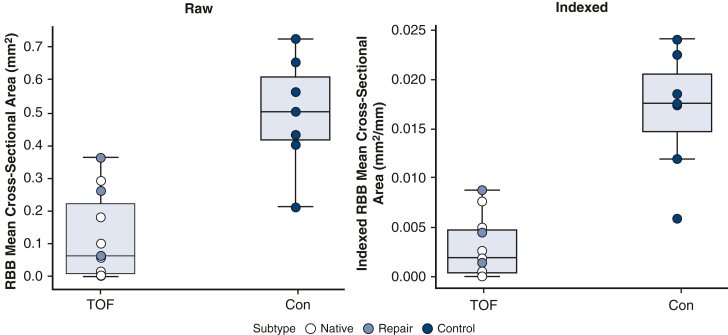
Right bundle branch (RBB) mean cross-sectional area in tetralogy of Fallot (TOF) and control (Con) hearts for raw measurements and values indexed to ventricular length. *Lower and upper borders of the box* represent the lower and upper quartiles (25th percentile and 75th percentile) with the median indicated by the *horizontal line*; *whiskers* extend to the minimum and maximum nonoutlier values. *White circles* represent native TOF hearts, *light-blue circles* repaired TOF hearts, and *navy circles* disease-free control hearts.

Overall, ToF hearts demonstrated significantly smaller RBB cross-sectional areas compared with controls (0.12 ± 0.13 mm^2^ [median, 0.063; range, 0.0001-0.36] vs 0.50 ± 0.17 mm^2^ [median, 0.50; range, 0.21-0.72]; *P* = .0005). When indexed to ventricular length, similar results were observed (3.0 ± 3.1 × 10^−3^ mm^2^/mm [median, 1.9; range, 0.003-8.8 × 10^−3^] vs 16.8 ± 6.2 × 10^−3^ mm^2^/mm [median, 17.5; range, 5.8-24.1 × 10^−3^]; *P* = .0006) (Figure 7).

### VR as a Clinically Relevant Output

The HiP-CT to VR pipeline was successfully implemented, as proof of concept, in all trialed datasets (10 ToFs, 1 control). VR allowed detailed exploration of the AVCS and reslicing of the data in any plane as a supplementary aid to understand the spatial relationship of the AVCS to key neighboring structures (MPM, ASC, and aortic valve), and to trace complex pathways in 3D (Video 2).

## Discussion

This first HiP-CT application to a pediatric congenital heart disease case series provides new 3D data concerning the ToF AVCS. HiP-CT delivers near-histologic resolution while preserving whole-organ spatial context and maintaining manageable data size, enabling 3D reconstruction and visualization of the entire heart and AVCS, with the capability for segmentation and examination in any plane. Such HiP-CT data have a potential use in hands-on surgical training (3D printing and XR), as a training set for machine learning, and for complex electrophysiologic simulations. Conventional histology relies on destructive methods and 3D reconstruction based on limited 2-dimensional data, risking anatomical misinterpretation or missing significant features.

Our findings corroborate previous studies regarding the anatomical position of the ToF NBB. The NBB is closely associated with the ASC and typically lies centrally or toward the left side of the IVS. In ToF with perimembranous VSDs, the NBB lies between the membranous and muscular septa.^7^ The NBB is superficial and therefore at risk during transatrial TV leaflet retraction or VSD closure: the region of fibrous continuity between the atrioventricular valves at the posteroinferior VSD margin is a recognized “danger” zone in PM defects.^7^^,^^8^^,^^10^ We show that conduction tissue lies within 1 to 3 mm of the membranous septum remnant. Suturing here therefore requires caution.

The left side of the septal crest was entirely draped by conduction tissue in all ToF specimens. In some, the BB draped into the RV below the crest. The RBB's variable origin, anywhere from the TV annulus to the VSD nadir, highlights the need for caution in this region. Using the intersection of the VSD with the aortic leaflets as a reference point, no branching was observed between this point and 2 mm beyond the start of the curvature of the VSD nadir. No correlation was found between RBB origin location and its subsequent course in ToF, further complicated by the inconsistent association of the RBB with the MPM. RBB depth from the endocardial surface at origin ranged from 0.25 to 2.5 mm, becoming shallower within 1 to 3 mm. Although variable depth endangers the ToF RBB during surgery, its narrow nature could be protective.

Suturing 3 to 4 mm below the IVS crest, between the upwards curvature and nadir, poses moderate risk. A balance between suturing inferiorly and maintaining stability of the VSD patch is important. Suturing tangentially forehand or backhand parallel to the IVS crest could reduce this risk.

The ToF RBB was less traceable than in controls, and in 2 samples, the RBB remained invisible even at 2.203 μm/voxel, possibly because the RBB branches more proximally into thin, sheet-like structures, which are challenging to trace even at the greatest resolution HiP-CT currently available. The ToF dead-end tracts were continuations of the BB, following the VSD curvature toward the aortic root. Limited descriptions of similar tracts in literature are primarily in structurally normal hearts and in 1 ToF case.^23^^,^^24^ In 1 sample, an atypical, sheet-like fasciculoventricular tract was observed running superiorly. Fasciculoventricular connections, ubiquitous in normal and congenitally malformed hearts, can create re-entry circuits, independent of AVN input. These can be insulated and bypass normal conduction pathways, enabling atypical forms of ventricular pre-excitation.^25^ The persistence of dead-end tracts may provide an arrhythmogenic substrate for focal triggered activity or re-entrant circuits, particularly within the outflow tract regions, leading to ventricular arrhythmias, as has been reported in structurally normal hearts.^26^ The persistence of abnormal tracts in ToF, accurately visualized by HiP-CT, appears more common than previously described and could represent an under-recognized substrate for spontaneous ventricular arrhythmias and unsuccessful ablation outcomes.^25^^,^^26^

Our observations of a less well-defined AVCS in ToF and additional anomalies suggests abnormal differentiation during development, potentially providing a larger substrate for both pre- and postoperative arrhythmias, driven by enhanced automaticity or triggered activity within the atrioventricular junction (eg, junctional ectopic tachycardia), or for ectopic impulse generation and re-entry. In many respects, the ToF AVCS resembles early stages of cardiac development, for example, at Carnegies stage 16/17 in the human embryo, before septal closure and remodeling of conduction tissue in relation to the IVS crest.^27^, ^28^, ^29^ These anomalies may reflect altered differentiation/remodeling of cardiomyocytes into conduction tissue, driven by key transcription factors which in ToF may be further impacted by subsequent RV remodeling.^30^^,^^31^ Collaborative work is currently underway to explore such mechanisms, combining spatial transcriptomic data with 3D volumes obtained through HiP-CT.

This study demonstrates proof of concept for a complete pathway integrating HiP-CT segmentations into VR. Although 3D reconstructions can be produced in specialized software, interpretation on 2-dimensional screens remains challenging. Here, VR was shown to be a useful aid for visualizing and manipulating complex cardiac anatomy, allowing the AVCS to be examined in relation to surrounding heart structures. In future, it may be possible in VR to train surgeons to avoid the AVCS in ToF through surgical or patch planning simulations. Further development and validation of the HiP-CT to VR pipeline is warranted to establish its clinical and educational utility.

This study shows that heterogeneity of the ToF AVCS makes its course unpredictable, highlighting the value of intraoperative conduction mapping. Successful localization of the NBB using high-density multielectrode catheters has been achieved and shows promise in reducing postoperative atrioventricular block.^32^^,^^33^ Expanding intraoperative mapping to detect the RBB could further reduce surgical risk.

### Limitations

Phase-contrast tomography at synchrotrons remains an expensive and limited-access technique, restricted to fixed ex-vivo imaging due to the need for absorption normalization, autoabsorption protocols, and the very high radiation dose. Furthermore, fixation may produce subtle changes to conduction tissue morphology (eg, millimeter depth from surface), which may differ from that present in-vivo or during surgery. Laboratory-based systems under development may broaden access. The relatively small and diverse sample set limited further subanalyses. Despite careful selection of intact specimens, handling over time occasionally caused unexpected fractures and fragmentation of conduction tissue not visible endocardially. The 2.203-μm/voxel “zooms” did not always resolve distal conduction tissue. Greater resolution might amplify noise rather than signal. A major bottleneck is the segmentation process, which is time-consuming and requires expert morphologists. The development of machine learning models for automatic segmentation is currently underway in our group, using ground truth data from this study.

## Conclusions

Using HiP-CT imaging of 18 whole hearts (11 ToF, 7 controls), coupled with custom morphometric pipelines and VR visualization, we demonstrate significant heterogeneity of the ToF conduction system, including variable RBB origin, depth, and hypoplasia; draped and immature conduction morphology; the presence of dead-end tracts and fasciculoventricular connections; and define precise regions of surgical vulnerability around the VSD. These findings redefine the anatomic substrate in ToF and have direct relevance for defining preoperative risk, informing surgical techniques, and demonstrating the importance of intraoperative conduction mapping strategies.

## Conflict of Interest Statement

The authors reported no conflicts of interest.

The *Journal* policy requires editors and reviewers to disclose conflicts of interest and to decline handling or reviewing manuscripts for which they may have a conflict of interest. The editors and reviewers of this article have no conflicts of interest.

## Figures and Tables

**Table E1:** Scanning parameters for each HiP-CT scan

Sample ID	Voxel size (um)	Data DOIs	Propagation distance (m)	Attenuator	Mean energy (keV)	Lateral field of view (mm)	Projections	Exposure time (ms)	Accumulation	Acquisition mode	Scan time (s/scan)	Total number of scans	Vertical field of view/scan (mm)	Vertical translation
125	9.595	http://doi.org/10.15151/ESRF-DC-2299323256	10	Sapphire 5 mm Mo 0.21 mm	96	81.14	12,000	20	1	Half-acquisition	265	15	8.16	7
	2.21	http://doi.org/10.15151/ESRF-DC-2299328558	1	Sapphire 5 mm, sapphire 5 mm, Ag 0.1 mm	86.6	13.44	15,000	20	1	Half-acquisition	107	17	2.94	1.5
173	9.595	http://doi.org/10.15151/ESRF-DC-2298042950	10	Sapphire 5 mm Mo 0.21 mm	96	81.14	12,000	20	1	Half-acquisition	265	14	8.16	7
	2.203	http://doi.org/10.15151/ESRF-DC-2299328589	1.6	Sapphire 5 mm, Ag 0.2 mm	89.6	19.95	15,000	35	1	Half-acquisition	616	5	6.52	4
185	9.595	http://doi.org/10.15151/ESRF-DC-2298042926	10	Sapphire 5 mm, Mo 0.21 mm	96	81.14	12,000	20	1	Half-acquisition	265	15	8.16	7
	2.203	http://doi.org/10.15151/ESRF-DC-2299328595	1.6	Sapphire 5 mm, Ag 0.2 mm	89.6	19.95	15,000	35	1	Half-acquisition	618	1	6.62	4
450	9.595	http://doi.org/10.15151/ESRF-DC-2298042944	10	Sapphire 5 mm Mo 0.21 mm	96	81.14	12,000	20	1	Half-acquisition	265	18	8.16	7
	2.203	http://doi.org/10.15151/ESRF-DC-2299328601	1.6	Sapphire 5 mm, Ag 0.2 mm	89.6	19.95	15,000	35	1	Half-acquisition	616	6	6..52	4
727	9.595	http://doi.org/10.15151/ESRF-DC-2299337101	10	Sapphire 5 mm, Mo 0.21 mm	96	81.14	12,000	20	1	Half-acquisition	265	16	8.16	7
	2.203	http://doi.org/10.15151/ESRF-DC-2299335007	1.6	Sapphire 5 mm, Ag 0.2 mm	89.6	19.95	15,000	35	1	Half-acquisition	618	1	6.52	4
769	20.01	http://doi.org/10.15151/ESRF-DC-2299323263	10	Sapphire 10 mm, Ag 0.2 mm, C 25 mm	108	117.9	12,000	10	4	Quarter-acquisition	530	15 × 2	8.00	7
	2.203	http://doi.org/10.15151/ESRF-DC-2299335013	1.6	Sapphire 5 mm, Ag 0.2 mm	86.2	19.95	15,000	40	1	Half-acquisition	704	6	6.52	4
1770	9.595	http://doi.org/10.15151/ESRF-DC-2298042938	10	Sapphire 5 mm Mo 0.21 mm	96	81.14	12,000	20	1	Half-acquisition	265	15	8.16	7
	2.203	http://doi.org/10.15151/ESRF-DC-2299328576	1	Sapphire 10 mm, Ag 0.1 mm	86.6	19.95	15,000	20	1	Half- acquisition	4030	10	3.09	1.5
1771	9.595	http://doi.org/10.15151/ESRF-DC-2299337089	10	Sapphire 5 mm Mo 0.21 mm	96	81.14	12,000	20	1	Half-acquisition	265	15	8.16	7
	2.203	http://doi.org/10.15151/ESRF-DC-2299335019	1.6	Sapphire 5 mm, Ag 0.2 mm	89.6	19.95	15,000	35	1	Half-acquisition	616	6	6.52	4
1840	19.19	http://doi.org/10.15151/ESRF-DC-2298042932	12	Sapphire 5 mm Mo 3.75 mm SiO2 bars 25 mm	155	137	12,000	13	3	Quarter-acquisition	516	21 × 2	8.16	7
	2.203	http://doi.org/10.15151/ESRF-DC-2299335025	1.6	Sapphire 10 mm, Ag 0.1 mm	86.2	19.95	15,000	40	1	Half-acquisition	704	12	6.52	4
2010	19.19	http://doi.org/10.15151/ESRF-DC-2299479757	12	Sapphire 5 mm Mo 3.75 mm SiO2 bars 25 mm	155	137	12,000	13	3	Quarter-acquisition	516	16 × 2	8.06	7
	2.203	http://doi.org/10.15151/ESRF-DC-2299335031 and http://doi.org/10.15151/ESRF-DC-2299337083	1.6	Sapphire 10 mm, Ag 0.1 mm	86.2	19.95	15,000	40	1	Half-acquisition	699	9	6.52	4
2577	10.005	http://doi.org/10.15151/ESRF-DC-2299323270	10	Sapphire 10 mm Ag 0.2 mm C 20 mm	105	90.6	12,000	20	1	Half-acquisition	265	16	8.00	7
	2.203	http://doi.org/10.15151/ESRF-DC-2299335037	1.6	Sapphire 5 mm, Ag 0.2 mm	89.6	19.95	15,000	35	1	Half-acquisition	617	1	6.52	4
2359	9.595	http://doi.org/10.15151/ESRF-DC-2299337095	10	Sapphire 5 mm Mo 0.21 mm	96	81.14	12,000	20	1	Half-acquisition	265	14	8.16	7
2337	10.005	http://doi.org/10.15151/ESRF-DC-2299323283	10	Sapphire 10 mm Ag 0.2 mm C 20 mm	108	90.6	15,000	20	3	Half-acquisition	1026	15	8.9	7
2625	7.032	https://doi.esrf.fr/10.15151/ESRF-DC-2406973754 and https://doi.esrf.fr/10.15151/ESRF-DC-2406973754	7	Sapphire 10 mm, Ag 0.2 mm, glassy carbon 30 mm	105	66.5	15,000	20	3	Half-acquisition	1023	11	8.02	7
2627	7.032	http://doi.org/10.15151/ESRF-DC-2299323277	7	Sapphire 10 mm, Ag 0.2 mm, glassy carbon 30 mm	105	66.5	15,000	20	3	Half-acquisition	1027	12	8.02	7
	2.203	http://doi.org/10.15151/ESRF-DC-2299335044	2.1	Sapphire 10 mm, Ag 0.2 mm, glassy carbon 30 mm	94.5	19.95	15,000	50	1	Half-acquisition	871	1	6.52	4
2628	7.032	http://doi.org/10.15151/ESRF-DC-2299323289	7	Sapphire 10, Ag 0.3 mm, glassy carbon 30 mm	105	66.5	15,000	20	3	Half-acquisition	1027	12	8.02	7
2629	7.032	http://doi.org/10.15151/ESRF-DC-2299323295	7	Sapphire 10 mm, Ag 0.2 mm, glassy carbon 30 mm	105	66.5	15,000	20	3	Half-acquisition	1027	9	8.02	7
	2.201	http://doi.org/10.15151/ESRF-DC-2299335050	2.1	Sapphire 10 mm, Ag 0.3 mm, glassy carbon 9.6 mm	94.5	19.95	15,000	50	1	Half-acquisition	872	1	6.52	4
2630	7.032	http://doi.org/10.15151/ESRF-DC-2299323301	7	Sapphire 10 mm, Ag 0.2 mm, glassy carbon 30 mm	105	66.5	15,000	20	3	Half-acquisition	1027	9	8.02	7

*HiP-CT*, Hierarchical phase-contrast tomography.

**Table E2:** Atrioventricular conduction system structures visible in original whole-organ dataset (checkmarks) and in local high-resolution zoom scans 2.21 μm/2.203 μm (italicized checkmarks)

Sample	AVN	PB	LBB	RBB origin	RBB	NBB
125	✓	✓	✓	✓	✓	*✓*
173	✓	✓	*✓*	*✓*	*✓*	*✓*
185	✓	✓	✓	✓	*✓*	*✓*
450	✓	✓	✓	✓	*✓*	*✓*
727	✓	✓	*✓*	*✓*	*✓*	*✓*
769	✓	✓	✓	✓	✓	✓
1770	✓	✓		*✓*	*✓*	*✓*
1771	✓	✓	✓	✓	*✓*	*✓*
1840	✓	✓	*✓*	*✓*	*✓*	*✓*
2010	✓	✓	✓	*✓*	*✓*	*✓*
2577	✓	✓	*✓*	*✓*	*✓*	*✓*
2359	✓	✓	✓	✓	✓	✓
2337	✓	✓	✓	✓	✓	✓
2625	✓	✓	✓	✓		
2627	✓	✓	✓	✓	*✓*	*✓*
2628	✓	✓	✓	✓	✓	✓
2629	✓	✓	✓	✓	✓	✓
2630	✓	✓	✓	✓	✓	✓

*AVN*, Atrioventricular node; *PB*, penetrating bundle; *LBB*, left bundle branch; *RBB*, right bundle branch; *NBB*, nonbranching bundle.

## APPENDIX E1

### Sample Preparation and Data Acquisition

Each heart was mounted in a container filled with 4% neutral buffered formalin and agar to stabilize the sample and prevent motion during acquisition. To minimize the formation of bubbles caused by radiation exposure, specimens underwent in-line degassing before imaging. The containers were then hermetically sealed until scanning started.

A filtered parallel polychromatic X-ray beam was used with a 178-m source-to-sample distance and propagation distances of 10 m (overview) and 1 m/1.6 m (local zoom).^E1^ X-rays were converted to visible light using LuAG:Ce scintillators (Crytur, Czechia) of 2000 μm (overview) or 50- to 100-μm (zoom) thickness, demagnified by Dzoom optic (overview) or magnified by a fixed ×2 optic (zooms), and detected by an Iris 15 camera (Teledyne Photometrics). Specimens were scanned entirely at voxel sizes of 19.19 μm (tetralogy of Fallot [TOF] with lungs), 10 μm (ToF without lungs, and Zayed Centre for Research normal specimens), and 7.032 μm (Birmingham Children's Hospital normal controls), followed by local “zoom” scans in regions of interest at 2.2-μm voxel resolution in all ToF samples and 3 control samples. The difference in overview pixel sizes is related to the size of the specimen. For each acquisition, a scan of a sealed container filled with 4% agar/formalin with the same parameters to serve as a beam reference for flat-field correction and correct low-frequency artefacts. Full scan parameters are in Table E1.

### Data Processing and Analysis

Data reconstruction was performed using tomography processing software (night-rail,^E2^ Nabu^E3^) developed at European Synchrotron Radiation Facility, following the hierarchical phase-contrast tomography processing protocol as described by Brunet and colleagues.^E1^ The night-rail framework integrates normalization, reference pairing, vertical concatenation (or helical). Ring artifact correction was applied on the reconstructed slices using an updated version of the Lyckegaard algorithm.^E4^ A 2-dimensional unsharp mask was applied to enhance contrast. Processing and visualization of the large datasets required high-performance computing resources.^E1^

Images were preprocessed by optimizing image brightness and contrast. 16-bit TIFF stacks were converted to 8-bit using ImageJ v1.54g^E5^ to reduce size and processing time. Conduction tissue was identified for segmentation following the rules of Aschoff and Monckeberg and promoted by Anderson and colleagues^E6^: conduction myocytes were histologically discrete from working myocardium, serially traceable section to section, and insulated by a fibrous sheath. The nonbranching bundle (of His) was first located in a 4-chamber plane, where it is characteristically surrounded by connective tissue, connecting inferiorly to the atrioventricular node and superiorly to the branching bundles. In Amira (2023.2), an unsharp mark and thresholding were applied to enhance tissue definition. Conduction tissue was manually segmented every fifth slice and interpolated using an in-house ImageJ macro (see Code Availability). Three-dimensional renderings were generated in Amira 2023.2 and VGStudioMax 2024.2.

## Appendix E1

Video 1 3D representation of a ToF conduction system as visualized by HiP-CT imaging, rendered in VGStudioMax. *ToF*, Tetralogy of Fallot; *HiP-CT*, hierarchical phase-contrast tomography *VSD*, ventricular septal defect; *AVN* atrioventricular node; *NBB*, non-branching bundle; *TV*, tricuspid valve; *RBB*, right bundle branch; *LBB*, left bundle branch. Video available at: https://www.jtcvs.org/article/S2950-6050(26)00015-X/fulltext.

Video 2 3D representation of a disease-free control specimen as visualized by VheaRts software, demonstrating the ability to rotate, slice and visualise the model in any plane. Video available at: https://www.jtcvs.org/article/S2950-6050(26)00015-X/fulltext.
