# Supplementary figures and images for: The heterogeneous nature of atrioventricular conduction tissues in tetralogy of Fallot demonstrated by hierarchical phase-contrast tomography
Source: JTCVS Struct Endovasc. 2026 Mar 20;10:100111. doi: 10.1016/j.xjse.2026.100111 (PMC13244724; doi:10.1016/j.xjse.2026.100111)

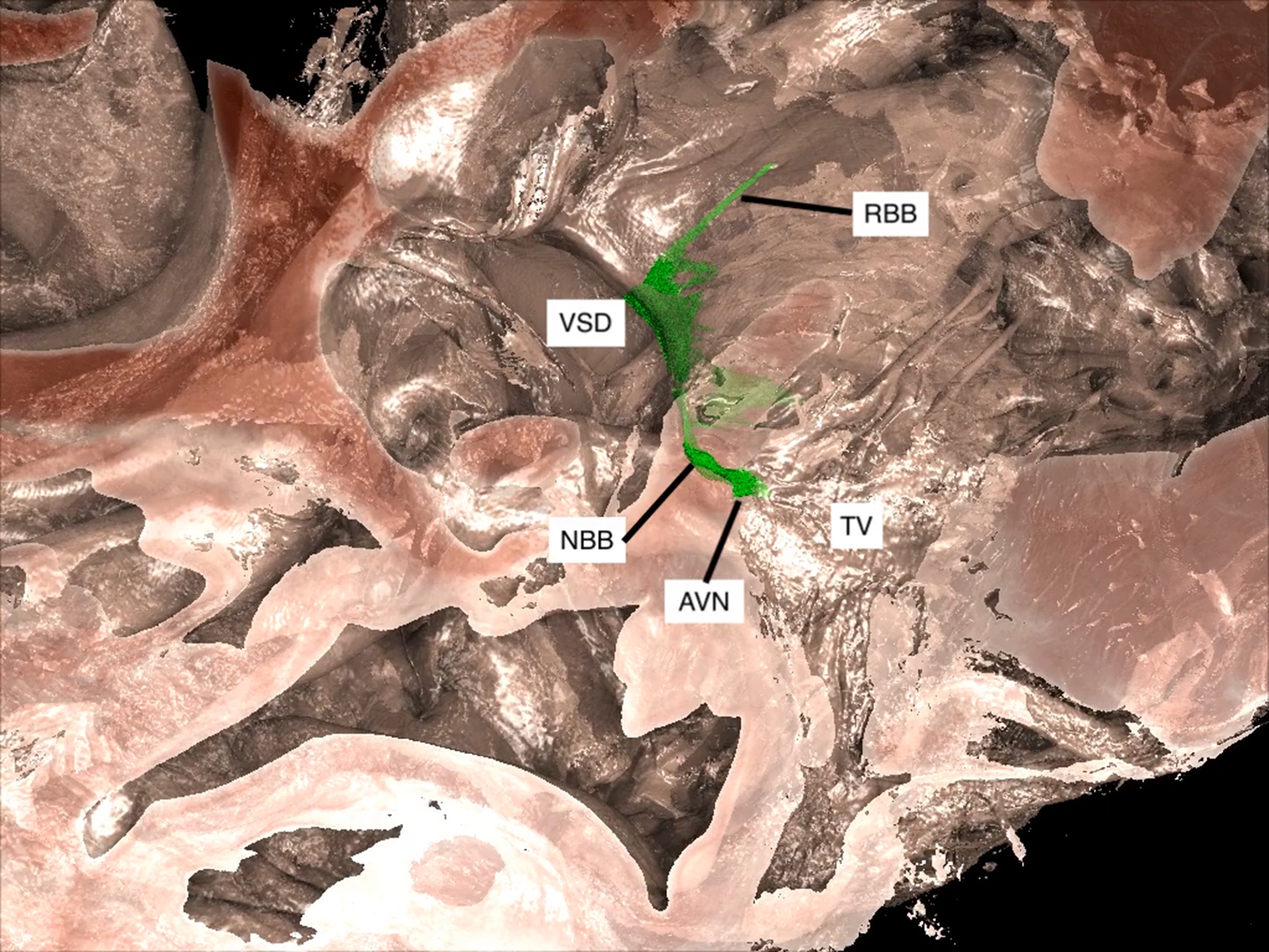

Supplement: Video 1 — 3D representation of a ToF conduction system as visualized by HiP-CT imaging, rendered in VGStudioMax. ToF, Tetralogy of Fallot; HiP-CT, hierarchical phase-contrast tomography VSD, ventricular septal defect; AVN atrioventricular node; NBB, non-branching bundle; TV, tricuspid valve; RBB, right bundle branch; LBB, left bundle branch. Video available at: https://www.jtcvs.org/article/S2950-6050(26)00015-X/fulltext. [file fx2.jpg]

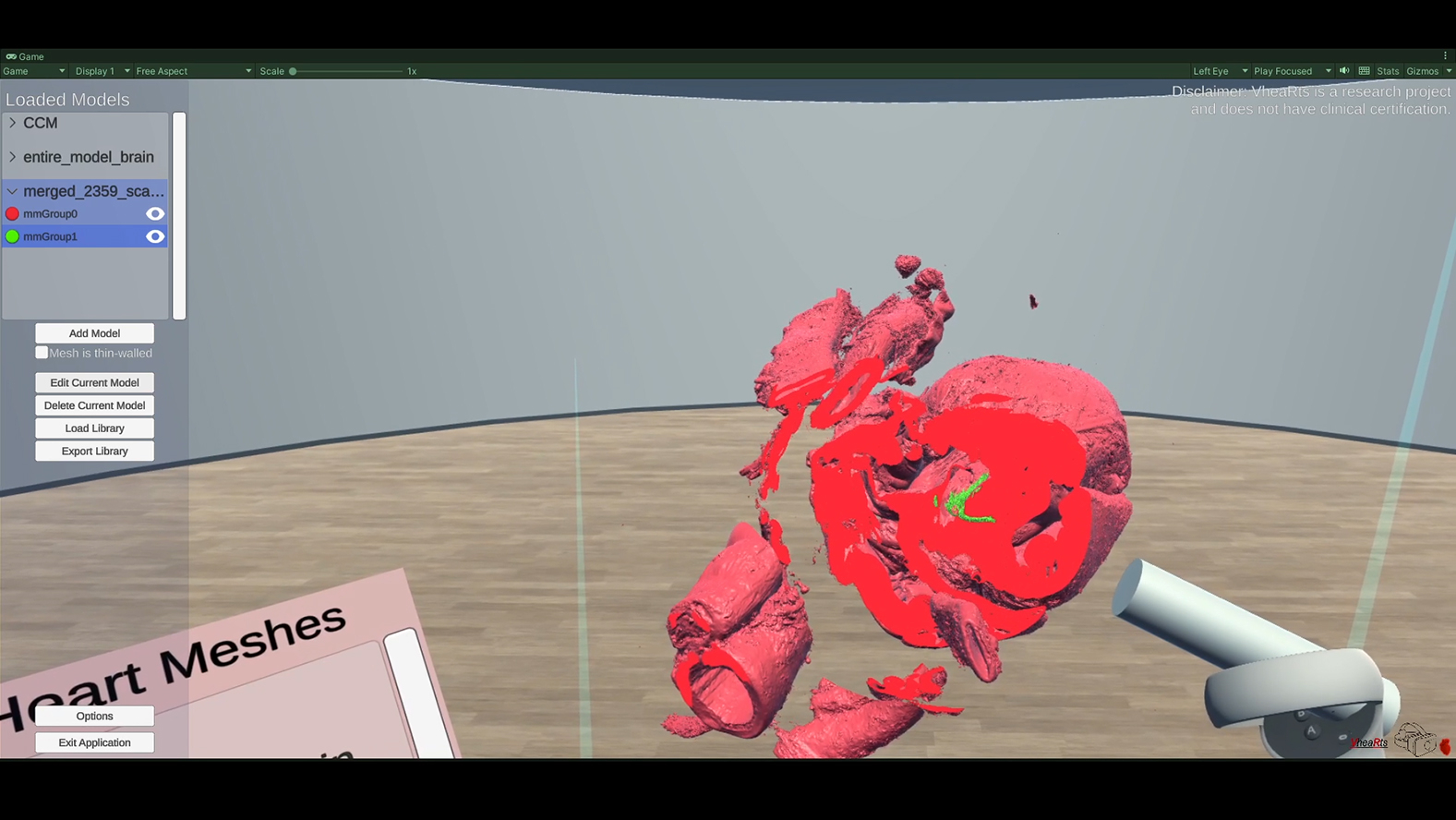

Supplement: Video 2 — 3D representation of a disease-free control specimen as visualized by VheaRts software, demonstrating the ability to rotate, slice and visualise the model in any plane. Video available at: https://www.jtcvs.org/article/S2950-6050(26)00015-X/fulltext. [file fx3.jpg]
